# Light-Driven Changes in Macrophyte Tissue Quality Affect the Composition of Associated Microbial Communities

**DOI:** 10.1007/s00248-025-02546-9

**Published:** 2025-05-23

**Authors:** Mandy Velthuis, Luca Zoccarato, Annelies J. Veraart, Michael T. Monaghan, Elisabeth Funke, Piet Verdonschot, Hans-Peter Grossart, Sabine Hilt

**Affiliations:** 1 Leibniz Institute of Freshwater Ecology and Inland Fisheries (IGB), Berlin, Germany; 2 Wageningen University and Research, Wageningen, the Netherlands; 3 Netherlands Institute of Ecology (NIOO-KNAW), Wageningen, the Netherlands; 4 Vienna, Core Facility Bioinformatics, University of Natural Resources and Life Sciences, Muthgasse 18, 1190 Vienna, Austria; 5 Vienna, Department of Biotechnology, Institute of Computational Biology, University of Natural Resources and Life Sciences, Muthgasse 18, 1190 Vienna, Austria; 6 Department of Ecology, Radboud Institute for Biological and Environmental Sciences, Radboud University, P.O. Box 9010, 6500 GL Nijmegen, the Netherlands; 7 Institut Für Biologie, Freie Universität Berlin, Berlin, Germany; 8 University of Amsterdam, Amsterdam, the Netherlands; 9 Potsdam University, Potsdam, Germany

**Keywords:** Bacterial 16S, Ecological stoichiometry, *Elodea nuttallii*, Fungal LSU, Macrophyte decomposition, Phenolic content, Epiphytic biofilm

## Abstract

Microbial biofilms are important components in macrophyte decomposition, and their composition depends on the decomposition stage and host plant quality. Here, we investigated how macrophyte tissue quality (i.e., C:N:P stoichiometry and phenolic contents) influences epiphytic microbial biofilms during litter decomposition. Consecutive experiments were conducted to (1) modify the C:N:P stoichiometry and phenolic content of the freshwater macrophyte *Elodea nuttallii* by manipulating light and nutrient availability and (2) test how the modified tissue quality affected epiphytic microbial biofilm diversity and community composition before and during macrophyte decomposition. Our results showed that shading led to lower C:N ratios (28.6 to 12.6) and higher phenolic content (10.8 to 19.2 µg/mg dry weight). Simultaneously, shading affected the epiphytic bacterial and fungal community composition, and these shifts correlated with the macrophyte C:N ratio. While no effects of macrophyte tissue quality on decomposition rates were observed, the epiphytic bacterial community composition on the litter was significantly affected by light treatment, time, and their interaction. Bacterial community composition shifted from a high abundance of Comamonadaceae to a more diverse community over time. Overall bacterial diversity was lower on the litter grown in the shaded mesocosms. Fungal diversity and community composition during litter decomposition were not affected by litter quality. Overall, our results reveal a structuring role of macrophyte tissue quality on its associated microbial biofilm and uniquely show a continuation of light-driven changes in epiphytic bacterial community composition after exposure. We conclude that light-driven changes in C:N stoichiometry are a crucial factor in shaping epiphytic microbial communities during macrophyte decomposition.

**Supplementary Information:**

The online version contains supplementary material available at 10.1007/s00248-025-02546-9.

## Introduction

Submerged macrophytes are key elements of organic carbon (C) cycling in freshwater ecosystems [23]. Decomposition of macrophyte detritus is relevant for both C burial [7] and greenhouse gas emissions [16]. The relationships between microbial communities in freshwater ecosystems and the functioning of those systems are often closely intertwined [26]. Epiphytic microbial biofilms, for instance, are known to affect decomposition of macrophyte detritus [51]. Existing studies of epiphytic microbial biofilms focus primarily on bacterial and algal components [1, 26], and more recently, on their fungal community component [13, 51]. A large variety of bacterial classes are represented in epiphytic biofilms [21]. Generally, bacterial diversity is higher on macrophytes than in the surrounding water [26, 50]. Biofilms can also host a diverse community of fungi [47], and while these organisms play an important role in macrophyte decomposition [51], their community composition and ecology has been less frequently studied.

Microbial diversity and community composition of epiphytic biofilms are highly variable, and dependent on time, environmental conditions, as well as organic matter composition of the host plant [47]. Environmental stress can have a destabilizing effect on microbial communities, leading to decreased diversity [22]. Macrophytes can contain and exude polyphenols [15], which can have antimicrobial properties [5, 30] and alter the bacterial community composition of macrophyte biofilms [21]. In a similar manner, the elemental composition of primary producers in general is often used as a food quality predictor for higher trophic levels [42]. Bacterial assemblages in lakes are relatively phosphorus (P)-rich compared to environmental concentrations [41], indicating that macrophytes with a low C:P ratio could pose a more suitable environment for bacterial growth. Similar investigations on the role of macrophyte stoichiometry and metabolites for epiphytic fungal diversity are lacking for freshwater habitats.

Both the elemental and the polyphenolic content of freshwater macrophytes can vary depending on their environmental conditions. For instance, macrophyte C:N and C:P ratios are dependent on environmental nutrient availability [2, 45]. The effect of light availability on macrophyte tissue stoichiometry is debated in the scientific literature with examples of increased [17] and decreased [10] C:N ratios under shaded conditions, as well as no obvious light effects on macrophyte tissue stoichiometry [6]. Phenolic content can vary significantly between and within macrophyte species [24, 40] and are among other environmental variables dependent on light availability [17]. How light- and nutrient-driven changes in macrophyte quality influence structure and functions of microbial biofilms on living and decaying macrophytes remains to be investigated.

Here, two consecutive experiments were conducted to (1) modify the C:N:P stoichiometry and phenolic content of a submerged macrophyte by a full factorial combination of light and nutrient availability and (2) test how the modified tissue quality affected microbial macrophyte decomposition under field conditions. The freshwater macrophyte *Elodea nuttallii* was chosen as a model species due to the widespread occurrence in its native (North America) and invasive (most European countries, China, Japan, Türkiye, and the Philippines) range where it can form large monospecific stands with high biomass [9, 34, 49], its flexible tissue nutrient stoichiometry [45], and the presence of phenolic substances in its tissue [15]. In our experiments, the potential effects of the modified tissue quality on the diversity of the epiphytic microbial biofilm on *E. nuttallii* were evaluated. We hypothesized in the first experiment that tissue carbon (C):nitrogen (N) and C:P ratios decrease under high nutrient availability, while phenolic content is elevated under high light conditions. Under low light conditions, macrophytes may need to invest in more N-rich chlorophyll molecules [4], thus leading to decreased C:N ratio. We hypothesized in the second experiment that decomposition rates of *E. nuttallii* decrease with increasing carbon:nutrient ratios as well as with increasing phenolic content. Additionally, we anticipated that differences in the biofilm bacterial and fungal community composition and diversity during decomposition are related to macrophyte tissue quality. In general, microbial diversity is hypothesized to be impoverished under environmental stress(i.e., decreased light availability).

## Materials and Methods

### Experimental Modification of Macrophyte Tissue Quality

*E. nuttallii* was grown in outdoor mesocosms (66 L water and 24 L sediment) with distinct nutrient and light treatments. The two sediment nutrient treatments consisted of 10 and 100% pond soil and were achieved by mixing commercial pond soil (FloraGard Vertriebs-GmbH, Germany) and sand, according to Velthuis et al. [45] (Table S1). The mixed sediment was topped by a 1 cm layer of sand to provide a barrier for nutrient leaching from sediments into the water. The mesocosms were filled with tap water and half of them were covered with a green mesh shade cloth to achieve a light reduction of 78 ± 3.5% (mean ± SD) at the water surface (measured with a spherical PAR sensor (QSPL2101, Biospherical Instruments, USA)). The experiment was performed in a full-factorial design (*n* = 3) for a period of 2 months (from 15 June to 15 August 2018). To prevent excessive phytoplankton growth in the mesocosms, 5 L of water was refreshed twice a week from 26 th of July onwards. Detailed information on the experimental setup is included in the supplementary methodology.

After 2 months, the aboveground *E. nuttallii* biomass was harvested by cutting the stems just above the sediment surface. In the lab, the plants were carefully rinsed with tap water to remove any loosely attached algae. From each mesocosm, one plant tip (the top 5 cm) of a randomly selected *E. nuttallii* plant was weighed and freeze-dried for the determination of phenolic content, and one plant tip was weighed and frozen at − 80 °C for the analysis of microbial community composition. The remaining plant material, as well as the removed attached algae, was dried at 60 °C for 3 days and weighed. The plant material was then stored dry and in the dark for the decomposition experiment. The relative growth rate (RGR) was calculated as in Velthuis et al. [45].

The phenolic content was determined by the extraction of polyphenols from powdered, freeze-dried (1–4 mg) plant material [18]. The samples were incubated with 1 mL of 80% ethanol at 80 °C for 10 min and centrifuged for another 10 min at 10.000 rpm to separate the supernatant from the plant pellet. SDS/TEA solution (1% (w/v) and 5% (v/v), respectively) and a 0.01 M FeCl_3_ reagent were added to the supernatant in a 2:1 volume ratio. For the SDS/TEA solution, 2 g of sodium dodecyl sulfate (SDS) was dissolved in 190 mL demineralized water and mixed with 10 mL Triethanolamine (TEA). To create the ferrous chloride solution, 0.27 g of iron(III) chloride hexahydrate was dissolved in 100 mL 0.01 M HCl. Absorption at 510 nm was measured on a microtiter plate reader (BioTek, Bad Friedrichshall, Germany) using a tannic acid calibration curve as a reference. The P content was determined by combusting 1–2 mg of dry plant material at 550 °C for 30 min and converting all organic P to PO_4_^3−^ by digestion with a 2.5% (w/v) persulfate solution for 30 min at 121 °C. Thereafter, PO_4_^3−^ was measured colorimetrically on a QuAAtro39 Auto-Analyzer (SEAL Analytical Ltd. Southampton, UK). The C and N contents were determined on a Vario Micro Cube elemental analyzer (Elementar Langenselbold, Germany).

### Macrophyte Decomposition Under Field Conditions

Microbial decomposition of *E. nuttallii* aboveground biomass was determined using a litter bag method. Polyester litter bags of 6 × 10 cm and 300 µm mesh size were filled with 0.1 g of dried *E. nuttallii* biomass. These litter bags were placed in five 50 × 50 cm PVC-coated galvanized wire mesh cages with 2.5 cm mesh size (to exclude most herbivores and to facilitate retrieval at individual time points with limited disturbance of the remaining litter bags). Each cage contained 12 litter bags with *E. nuttallii* litter material. To correct for possible periphyton growth on and erosion of the litter bags, two empty litter bags were included in each cage as a control. The cages were deployed on the sediment of Lake Müggelsee at 2 m depth (52°26′51.4″ N 13°38′51.6″ E) on the 5 th of August 2019. After 7, 14, 28, 42, and 58 days, one of the cages was retrieved from the field location, and the litter bags were brought into the lab for further processing. Upon arrival in the lab, DNA samples were taken from the contents of the litter bags by swabbing them with a sterile cotton swab. This swab was flash frozen in liquid nitrogen and stored at − 80 °C until further processing for microbial sequencing analysis. Thereafter, the outside of the bags was rinsed with deionized water to remove any macroinvertebrates, and its contents were dried at 60 °C for 72 h and weighed. The mass loss of the litter bags with *E. nuttallii* biomass was corrected for the mass loss of the empty litter bags (0.01 ± 0.72%, *n* = 10).

### Community Composition and Diversity of the Microbial Biofilm

To quantify the microbial community grown on the plants in the mesocosms, frozen plants were swabbed with a sterile cotton swab, in a similar manner to the swabs from the litter bag samples as described above. DNA was extracted according to Nercessian et al. [31]. In short, each sample (i.e., the tip of the cotton swab) was mixed with 0.5 g of each 0.1 and 0.7 mm zirconia-silica beads, 750 µL extraction buffer (10% CTAB in 1.6 M NaCl and 240 mM potassium phosphate buffer at pH 8.0, mixed in a 1:1 ratio), 75 µL 10% sodium dodecyl sulfate, and 75 µL 10% N-lauroylsarcosin solution. To this mixture, 750 µL phenol–chloroform-isoamyl alcohol (25:24:1) was added, and the samples were vortexed for 35 s at 6.0 m/s (FastPrep-24™ 5G, Bio-Connect, Huissen, The Netherlands). After centrifugation at 14,000 g for 10 min at 4 °C, 500 µL of the upper phase was recovered and further mixed with 500 µL chloroform-isoamyl alcohol (24:1). After another round of centrifugation at 14,000 g for 10 min at 4 °C, the upper phase was mixed with 2 volumes of PEG buffer (30% PEG 6000 in 1.6 M NaCl) and 1 µL of LPA and incubated at room temperature in the dark for 2 h. After centrifugation at 17,000 g for 10 min at 4 °C, the precipitated DNA was recovered by removing the supernatant. This pellet was washed with 800 µL ice-cold 70% ethanol, centrifuged at 17,000 g for 10 min at 4 °C. After discarding the supernatant, the pellet was dissolved in 30 μL PCR water and stored at − 80 °C until further analysis.

For DNA samples from the growth experiment, PCR amplification (oligonucleotide primers in Table S2), library preparation, and sequencing (300-bp paired-end reads, Illumina Miseq v3 sequencing kit) of the bacterial 16S V5-V6 region of the rRNA gene and the fungal LSU rRNA gene regions were carried out by LGC Genomics GmbH (Berlin, Germany). For the decomposition experiment, bacterial 16S PCR amplification and sequencing-library preparation steps were performed at the Berlin Center for Genomics in Biodiversity Research on an automated workstation Biomek i7 hybrid (Beckman Coulter GmbH, Krefeld, Germany) in two PCR steps and using dual indexing, as described by Warter et al. [46]. Fungal LSU was PCR-amplified and sequenced as follows: a first PCR (96 °C for 30 s; 20 cycles of 96 °C for 30 s, 50 °C for 30 s, 72 °C for 60 s; 72 °C for 3 min) was carried out using 10 ng template DNA, 1 μL of each primer (10 µM), 2.5 μL mi-Taq Crystal buffer, and 0.2 μL polymerase (both Mi-Taq Only, Metabion International AG, Planegg/Steinkirchen, Germany), 0.5 μL dNTP-Mix and 0.25 μL 100 mM MgCl_2_ (Metabion International AG) and RO-filtered water for a total reaction volume of 25 μL. A second PCR (96 °C for 30 s; 20 cycles of 96 °C for 30 s, 55 °C for 30 s, 72 °C for 60 s; 72 °C for 5 min) was performed using 1 µL PCR product from the first step as a template. PCR products were purified using a magnetic bead protocol (Agencourt AMPure XP, Beckman Coulter, Indianapolis, IN, USA) following the manufacturer’s instructions. DNA concentration was measured using a Quantus fluorometer and the QuantiFluor dsDNA System (Promega, Madison, WI, USA), and all PCR products were normalized to a concentration of 5 ng/μL. An indexing PCR (95 °C for 2 min; 8 cycles of 95 °C for 20 s, 52 °C for 30 s, 72 °C for 30 s; 72 °C for 3 min) added unique 12-bp inline sequence barcodes (Nextera Index Kit, Illumina, San Diego, CA, USA) to each sample using 10 μL of PCR product mixed with 5 μL reaction buffer and 0.25 μL polymerase (both Herculase II Fusion DNA Polymerase, Agilent), 0.25 μL dNTP-Mix (25 mM each, Agilent), 0.625 μL each of index primers P5 and P7, and 1 μL DMSO and RO-filtered water. PCR products were purified twice and quantified as above. All samples were then pooled in equimolar amounts and sequenced (300-bp paired-end reads, Illumina MiSeq v3 sequencing kit, 600 cycles) at the Berlin Center for Genomics in Biodiversity Research. Due to insufficient fungal DNA yield from samples of day 14 and 28 of the light treatment, these were omitted from further analysis.

The resulting sequences were analyzed with the DADA2 bioinformatics workflow [8]. After visual inspection of the quality of the bacterial 16S sequences, forward reads were trimmed at a length of 230 and 280 bp for the growth and decomposition experiment, respectively, while reverse reads were all trimmed at a length of 200 bp, with maximum expected errors (maxEE) of 2 (function *filterAndTrim*). Fungal LSU sequences were trimmed with a minimal read length of 50 bp, maximum read length of 210 bp, and a maxEE of 2. The reads were denoised (function *dada*, *pool* = *TRUE*) and merged, and any chimeras were removed (function *removeBimeraDenovo*). Around 60% of raw reads were maintained and used for downstream analyses (Table S3). Taxonomy was assigned for the obtained amplicon sequence variants (ASVs) using Silva v138.1 and Eukaryome LSU v1.9.2 databases [36, 43]. As we were specifically interested in fungal LSU sequences, ASVs classified to other kingdoms were omitted from further analysis (52 and 37% of growth and decomposition sequences, respectively). Similarly, 16S ASVs that were classified as Archaea (< 1% for both growth and decomposition sequences) were omitted from further analysis. The accuracy of the taxonomic identification is summarized in Table S4.

### Statistics

All statistical analyses were carried out in R [37]. Plant parameters (relative growth rate, phenolic content, C:N:P stoichiometry, and elemental composition) were tested for effects of nutrient treatment, light treatment, and their interaction using two-way ANOVA models (function *aov*). Models were tested for normality by visual examination of the residuals, and data was transformed when necessary. Litter decomposition was described with a two-phase model [20] to obtain estimates for the decomposition rate *k* and recalcitrant fraction *s* (function *nlsLM*. Differences in the fraction of remaining dry weight in the litter bags over time were tested using a three-way ANOVA model (including nutrient treatment, light treatment, and time; function *aov*).

ASV diversity for both bacterial and fungal communities was calculated using the bias-corrected Chao1 index with the function *estimateR* from the vegan package [33] and tested for treatment effects in the same manner as the plant parameters. Differences in microbial community composition were assessed by testing the Bray–Curtis dissimilarity matrix for treatment effects with a Permutational Multivariate Analysis of Variance (PERMANOVA; *adonis2* function, vegan package). Furthermore, general patterns and clustering were assessed using distance-based redundancy analysis (dbRDA; function *capscale*, vegan package). In this analysis, Bray–Curtis distances were related to the nutrient and light treatments in a first model and to *E. nuttallii* quality characteristics (phenolic content, C:N, and C:P ratio) in a second model. The resulting models were tested for significance (*P* < 0.05) using a permutation test with 999 permutations (function *anova*).

## Results

### Experimental Modification of Macrophyte Growth and Tissue Quality

**Fig. 1:**
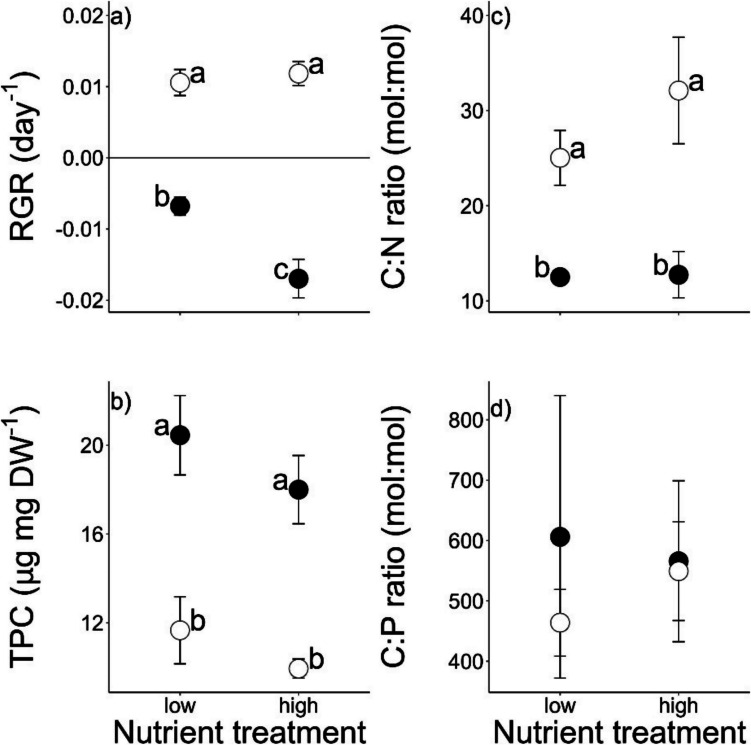
Growth and tissue quality (mean ± SE) of aboveground biomass of *Elodea nuttallii* grown in high and low sediment nutrient conditions in outdoor mesocosms, with (**a**) relative growth rate (RGR), (**b**) total phenolic content (TPC), (**c**) molar C:N ratio, and (**d**) molar C:P ratio. Light treatments include full light (◌) and shaded (●) conditions, and letters indicate significant pairwise differences

Throughout the growth experiment, mean (± SD) temperature was 19.66 ± 2.37 °C, pH 7.99 ± 0.23, and oxygen concentrations 8.32 ± 1.47 mg/L (see Table S5 for experimental conditions). Relative growth rates of *E. nuttallii* were significantly higher at high light availability (0.01 ± 0.003) compared to shaded conditions (− 0.01 ± 0.006, Fig. 1a, Table 1). There was a significant interaction between light and nutrient treatments (Table 1). In the high nutrient treatments, the negative effects of shading on macrophyte growth were stronger than under low nutrient conditions (Fig. 1a).

**Table 1:** Effects of nutrient treatment and light treatment and their interaction on growth, elemental composition, and phenolic content of *Elodea nuttallii* (two-way ANOVA). Bacterial (16S rRNA gene) and fungal (LSU rRNA gene) community composition was tested with a PERMANOVA test on Bray–Curtis distances

	*F* values		
Factor	Nutrient treatment	Light treatment	Nutrient * light treatment
*Plant parameters*			
Relative growth rate	5.29˙	141.19***	8.71*
Total phenolic content	2.18	35.43***	0.066
C:N	1.17	22.17**	1.02
C:P	0.14	0.08	0.10
C content	2.02	0.002	1.41
N content	0.004	48.69***	0.28
P content	0.025	0.022	0.003
*Microbial parameters*			
Bacterial ASV diversity	0.02	1.17	2.86
Fungal ASV diversity	2.98	2.54	0.08
Bacterial community composition	1.16	2.20***	1.14
Fungal community composition	0.90	2.13**	0.99

Significant *F*-values are indicated in bold, with ***P < 0.001, **P < 0.01, *P < 0.05, ˙*P* < 0.10

Phenolic content in macrophyte tissue was significantly higher under shaded conditions (19.2 ± 2.9 versus 10.8 ± 2.0 µg mg DW^−1^), while C:N ratios were significantly lower (12.6 ± 2.7 versus 28.6 ± 7.9; Fig. 1). Concurrent to the changes in C:N ratios, the N content of *E. nuttallii* was significantly higher under shaded conditions (6.77 ± 0.88 mmol g DW^−1^) compared to high light availability (3.09 ± 0.77 mmol g DW^−1^; Fig. S1). No effects of light treatment on C:P ratios, nor on C and P contents were observed. Furthermore, no effects of nutrient treatment on any of the measured growth or quality parameters of the plants were detected. The observed changes in phenolic content and C:N ratio were significantly correlated with each other (Fig. S2) in the sense that macrophyte tissues with high C:N ratios contained low phenolic content (*F*_(1,10)_ = 10.46, *R*^2^ = 0.46, *P* = 0.009).

### Linking Microbial Community Composition to Macrophyte Tissue Quality During the Growth Experiment

**Fig. 2:**
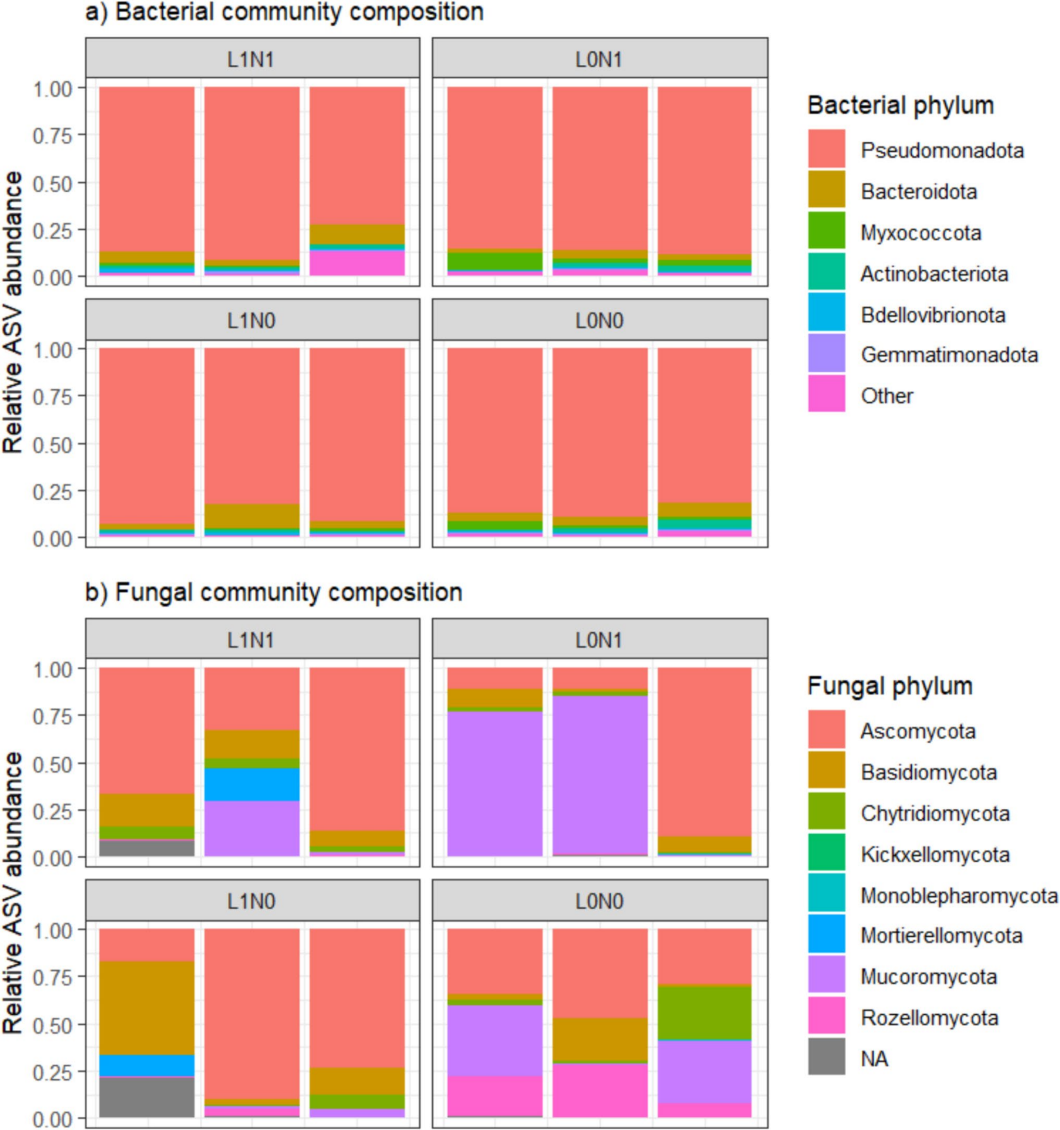
Relative abundance of the bacterial (**a**) and fungal (**b**) phyla in the *Elodea nuttallii* growth experiment. Each panel represents three replicates of a unique combination of nutrient and light treatment, with low (N0) and high (N1) nutrient treatments and shaded (L0) and full light (L1) treatments. Unknown sequences are indicated with NA. Bacterial phyla with a low abundance (< 0.5%) were grouped into the category “Other”

In the growth experiment, 1507 bacterial ASVs were retrieved from the biofilm of *E. nuttallii* and were classified as Pseudomonadota (55%), Bacteroidota (15%), Actinomycetota (7%), and Bdellovibrionota (6%) (Fig. 2a). The remaining sequences were allocated to various low-abundance (< 5%) bacterial phyla, with 1% of sequences not classified to any bacterial phylum. For the fungal community, 312 ASVs were revealed in our analysis and belonged to Ascomycota (34%), Basidiomycota (29%), Chytridiomycota (11%), and Mucoromycota (10%) (Fig. 2b). Remaining sequences belonged to various low-abundant fungal phyla (< 5%), and 11% of the sequences could not be assigned with the Eukaryome database. Microbial alpha diversity did not differ between nutrient or light treatments for both bacterial and fungal communities (Fig. S3, Table 1).

**Fig. 3:**
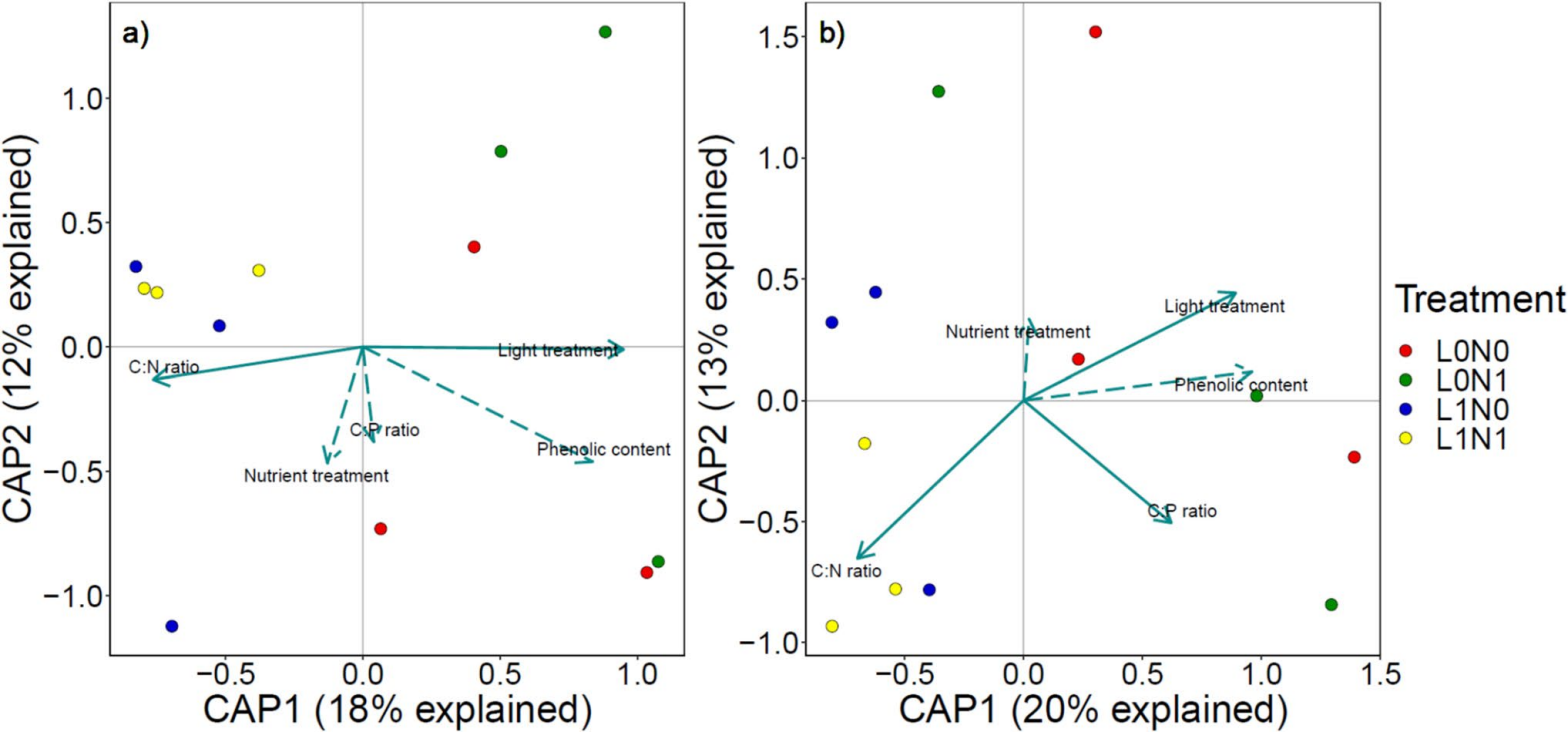
Distance-based Redundancy Analysis results of bacterial (**a**) and fungal (**b**) community composition on *Elodea nuttallii*. Significant parameters are indicated by solid arrows and different nutrient (high (N1) and low (N0)) and light (full light (L1), shaded (L0)) treatments are indicated by color

Potential differences between treatments in bacterial and fungal community composition were visualized and related to the environmental parameters measured in the growth experiment. For both bacterial and fungal datasets, light treatment significantly affected the community composition (*F*_(1,6)_ = 2.09, *P* = 0.004, and *F*_(1,6)_ = 2.33, *P* = 0.007, respectively), while no other measured environmental factors showed any correlation (Fig. 3). These results aligned with Permutational Multivariate Analysis of Variance (PERMANOVA) on Bray–Curtis distances, which showed significant differences in community composition between light treatments (Table 1). Under shaded conditions, the relative abundance of the bacterial family Methylophilaceae increased (Fig. S4). For the fungal community, relative abundance of Saksenaeaceae increased in the shaded treatment at the expense of Cladosporiaceae (Fig. S5). Interestingly, when experimental light and nutrient treatment were not included as explanatory factors, differences in bacterial community composition significantly correlated with *E. nuttallii* C:N ratio (*F*_(1,8)_ = 1.65, *P* = 0.04). Similarly, differences in fungal community composition showed significant correlations with *E. nuttallii* C:N ratio (*F*_(1,8)_ = 2.04, *P* = 0.006) and C:P ratio (*F*_(1,8)_ = 1.85, *P* = 0.02).

### Macrophyte Decomposition Under Field Conditions

Decomposition of *E. nuttallii* in eutrophic Lake Müggelsee occurred rapidly. The percentage of remaining biomass declined significantly over time, and no effects of light or nutrient treatment on this decline was observed (Fig. S6; Table 2). Across treatments, the percentage of recalcitrant material (*s*) was 17.9 ± 1.1% (mean ± SD). Decomposition rates *k* were on average 0.122 ± 0.007 day^−1^ across all treatments.

**Table 2:** Effects of nutrient treatment, light treatment, time, and their interactions on *Elodea nuttallii* decomposition and microbial diversity during the decomposition experiment in Lake Müggelsee (three-way ANOVA). Bacterial (16S rRNA gene) and fungal (LSU rRNA gene) community compositions were tested with a PERMANOVA test on Bray–Curtis distances

	*F* values				
Factor	%DW remaining	BacterialASVdiversity(log**-**transformed)	Bacterial community compositi**on**	Fungal ASV diversity (log-transformed)	Fungal community composition
Nutrient treatment	0.00	1.53	1.28	**3.47˙**	1.11
Light treatment	0.00	8.00**	7.44***	**3.39˙**	**1.60˙**
Time	57.04***	19.24***	23.53***	1.34	11.38***
Nutrient treatment * light treatment	0.20	0.56	1.24	0.82	1.14
Nutrient treatment * time	0.05	0.38	0.94	0.87	1.16
Light treatment * time	0.03	1.30	2.06*	1.35	1.37
Nutrient treatment * light treatment * time	0.01	1.39	0.76	0.46	1.06

Significant results are indicated in bold, with ***P < 0.001, **P < 0.01, *P < 0.05, ˙*P* < 0.10

### Biofilm Microbial Community Composition During Macrophyte Decomposition

**Fig. 4:**
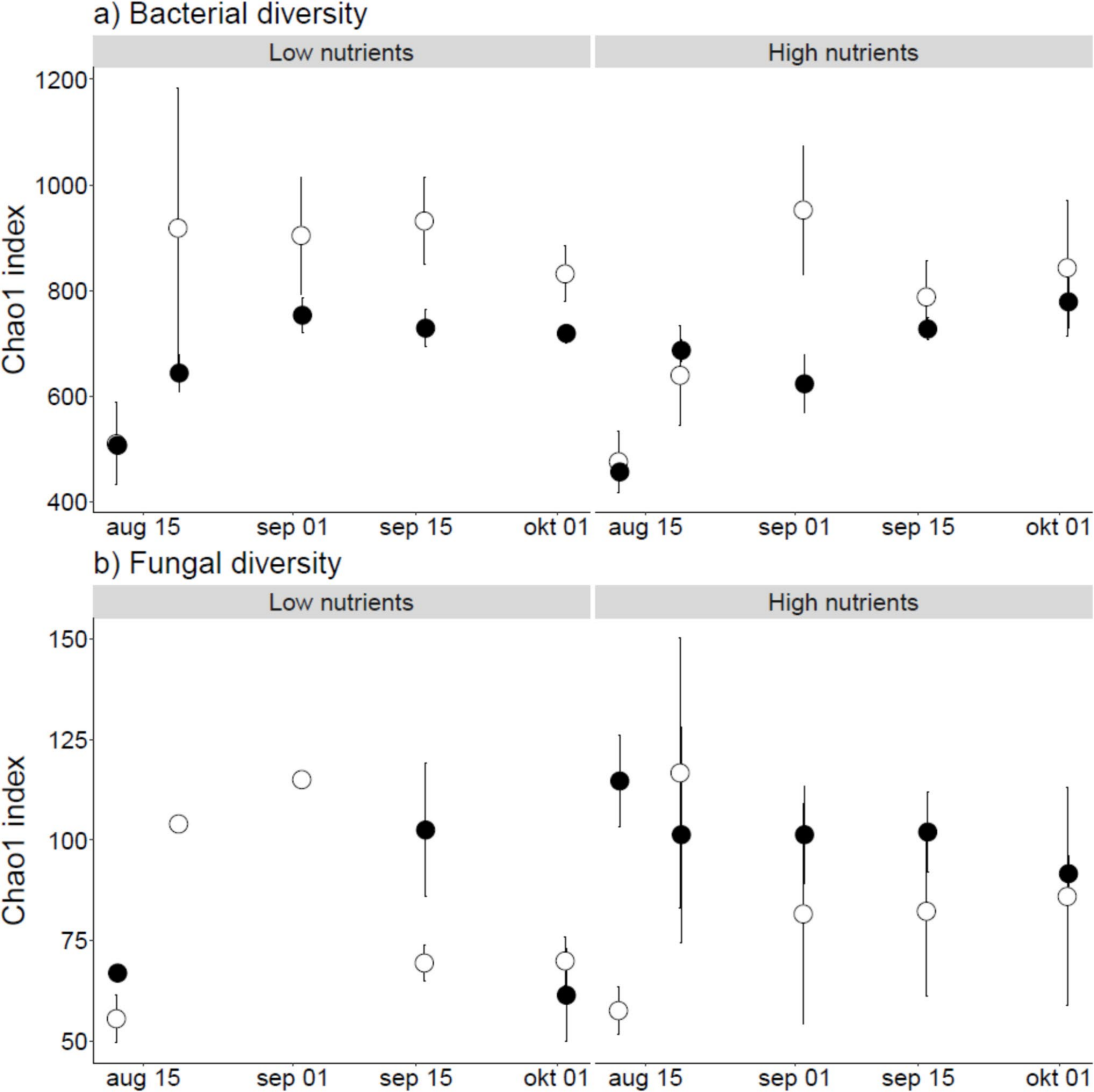
Bacterial (**a**) and fungal (**b**) alpha diversity, expressed as the bias-corrected Chao1 index (mean ± SE) over time during decomposition of *Elodea nuttallii* in Lake Müggelsee grown on low and high nutrient availability. Light treatments include full light (◌) and shaded (●) condition. Note that no fungal diversity data is available for day 14 and 28 in the light treatment due to insufficient DNA yield

**Fig. 5:**
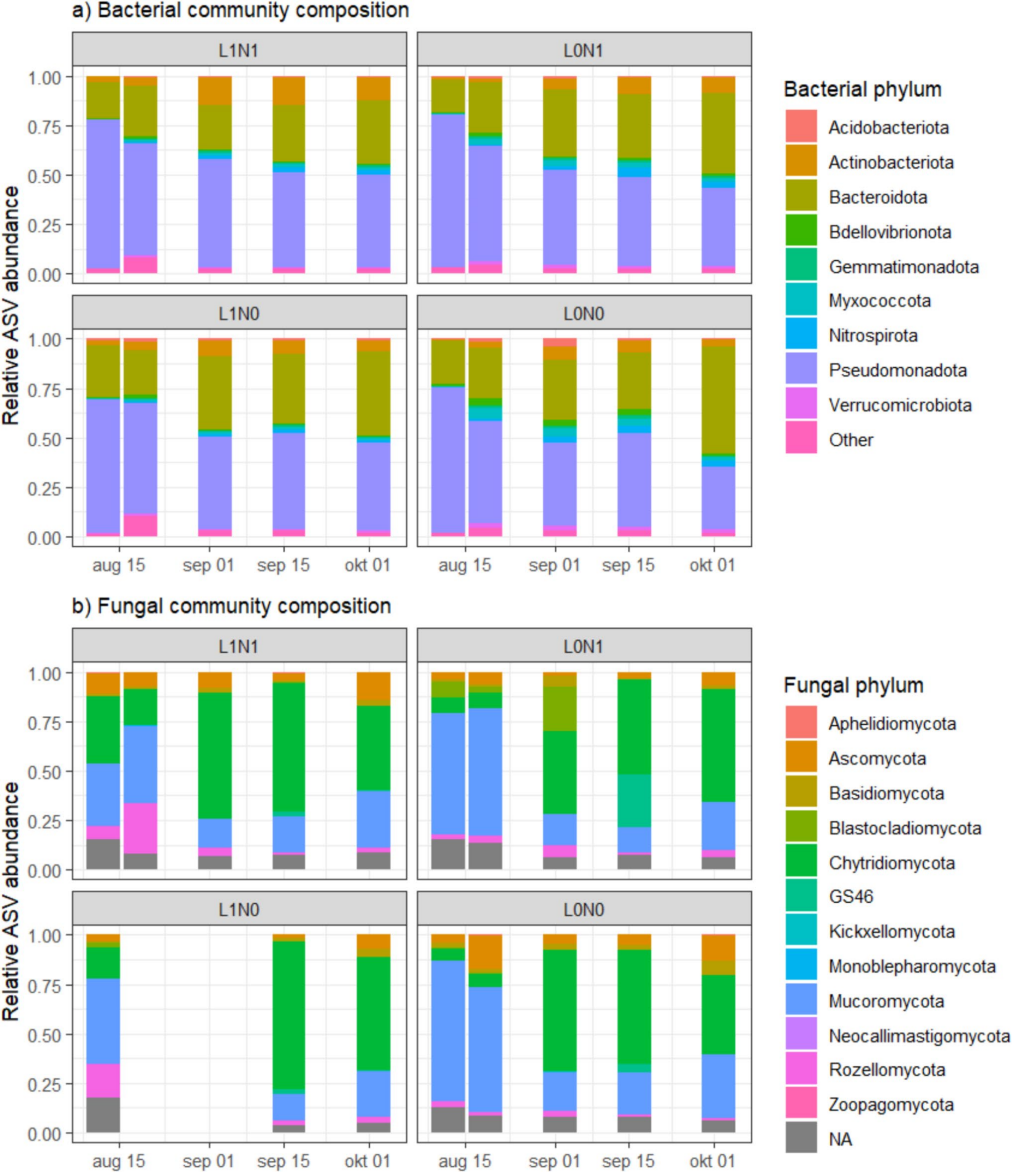
Mean relative abundance of bacterial (**a**) and fungal (**b**) phyla over time during decomposition of *Elodea nuttallii* in Lake Müggelsee (sample size varies between 1 and 3). Each panel represents a unique combination of nutrient and light treatment, with low (N0) and high (N1) nutrient treatments and shaded (L0) and full light (L1) treatments. Unknown sequences are indicated with NA. Note that no fungal community composition data is available for day 14 and 28 in the light treatment due to insufficient DNA yield

While no effects of light or nutrient treatment on macrophyte decomposition rates were observed under field conditions, bacterial ASV diversity significantly increased over time and was lower on the decomposing plants previously grown in the shaded mesocosm treatment (Table 2, Fig. 4a). The most abundant bacterial phyla were Pseudomonadota (40%), Bacteroidota (25%), Bdellovibrionota (8%), and Myxococcota (6%) (Fig. 5a). The remaining 21% of sequences were allocated to various non-abundant (< 5%) bacterial phyla.

**Fig. 6:**
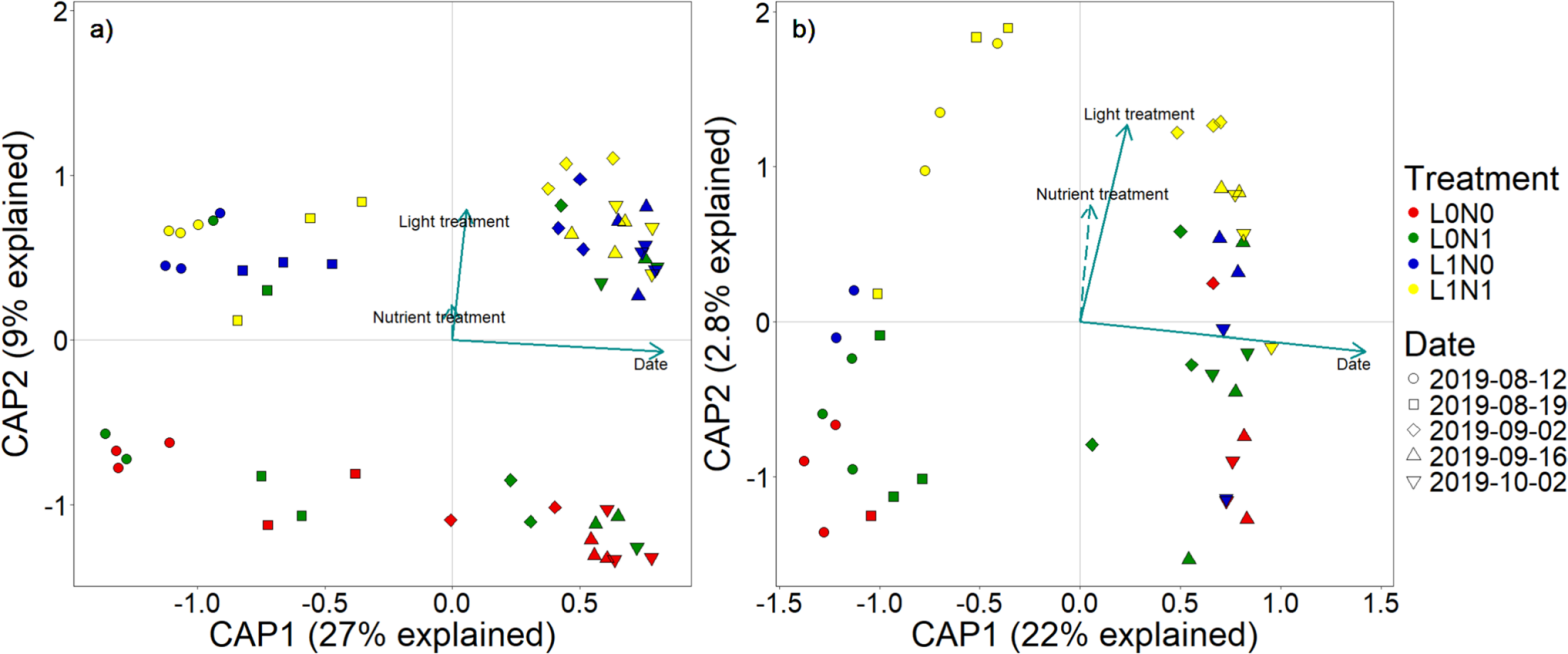
Distance-based Redundancy Analysis (dbRDA) results of bacterial (**a**) and fungal (**b**) community composition during the decomposition of *Elodea nuttallii*. Different nutrient and light treatments are indicated by color (see Fig. 3) and sampling dates by symbols. The first dbRDA axis is related to changes in community composition over time, whereas the effect of light treatment is visualized by the second dbRDA axis. Significant parameters (*P* < 0.10) are indicated by solid arrows. Note that light treatment was marginally significant (*P* = 0.08) in the fungal community composition (**b**)

Over time, the bacterial community composition shifted from a high abundance of Comamonadaceae (Pseudomonadota) to a more diverse bacterial community (Fig. 5a, Fig. S7). Patterns and clustering of the bacterial community composition were assessed using dbRDA (Fig. 6). The overall model was significant (*F*_(3,54)_ = 10.55, *P* = 0.001), and differences in bacterial community composition were significantly related to both light, sampling date, and their interaction (Table 2).

The fungal community composition during macrophyte decomposition was significantly affected by time and displayed a marginally significant effect of light treatment (Table 2, significant dbRDA model; *F*_(3,39)_ = 4.61, *P* = 0.001). The most abundant phyla, similar to the growth experiment, were Mucoromycota (29%), Chytridiomycota (21%), and Ascomycota (11%) (Fig. 5b). Twenty percent were allocated to various fungal phyla, and 19% of the sequences could not be assigned with the Eukaryome database. The initial community contained a high abundance of Saksenaeaceae (Mucoromycota), and the proportion of unknown sequences increased over time (Fig. 5b, Fig. S8). Fungal ASV diversity was not affected by nutrient treatment, light treatment, nor by sampling time (Table 2, Fig. 4b).

## Discussion

Understanding the effects of macrophyte tissue quality on their microbial decomposition is important, as quality can vary strongly depending on environmental conditions. In our experiments, light rather than nutrient availability altered macrophyte tissue nutrient stoichiometry and phenolic content, partially confirming our first hypothesis. As expected, the altered macrophyte tissue quality affected the fungal and bacterial community composition of epiphytic microbial biofilms. Surprisingly, no effects on macrophyte decomposition rates were observed. Lower light availability in freshwater ecosystems due to processes related to global change, such as eutrophication, brownification, or species invasions, may thus affect both macrophyte tissue quality and their associated microbial community structure. Ecosystem functioning such as macrophyte decomposition may still remain unchanged due to counteracting effects of tissue stoichiometry and polyphenols or functional redundancies in microbial communities.

### Macrophyte Tissue Quality in Response to Changes in Nutrient and Light Availability

Plants can adapt to low light conditions by investing more in N-rich chlorophyll compounds to enhance light acquisition [27]. In an experiment by Twilley and Barko [44], shaded conditions led to increased chlorophyll contents in three freshwater macrophytes. This may explain why shaded conditions led to increased N contents and decreased C:N ratios in *E. nuttallii*, though chlorophyll content was not measured. Under full light conditions, similar N content has been observed in a previous study using the same species [45].

In contrast to our hypotheses, C:N:P stoichiometry was unaffected by nutrient treatments, and shaded conditions led to increased phenolic content. These observations contradict previous research, where increased phenolic content under high light availability [17] and decreased C:P ratios with high nutrient availability [45] were observed. A possible explanation for this discrepancy could be the observed overall low growth rates of *E. nuttallii* in the experiment, and, in particular, the negative growth rates under the shaded conditions. These low growth rates could reduce stress by nutrient limitation and thereby avoid stoichiometric shifts. Similarly, growth stagnation under shaded conditions may have led to a relative buildup of phenolic content compared to the full light conditions, where plants may have invested in growth instead of secondary metabolite synthesis. The hypothesized light-driven increase of phenolic contents was based on studies in *Myriophyllum spicatum* [17] and the observed light-driven decrease in phenolic contents in *Elodea nuttallii* could thus indicate species-specific differences as well. In general, terrestrial literature suggests that slow-growing shade-tolerant plant species typically have a higher phenolic content [14], which fits with our experimental findings. Overall, our experimental results point to the dependence of macrophyte responses on variations in light and nutrient conditions and highlight the need for further research to better understand and predict phenolic acid synthesis in aquatic macrophytes.

### Effects of Macrophyte Tissue Quality on Decomposition

Previous studies have observed that both tissue stoichiometry and phenolic content of submerged macrophytes can affect their decomposition under field conditions. Faster decomposition rates are, for instance, reported for freshwater macrophytes with lower C:N ratios [3]. Alternatively, plants with higher phenolic content decompose slower, as illustrated, for instance, in salt marsh vegetation [48]. However, changes in tissue quality do not always lead to changes in decomposition rates, as Smith and Bradford [39] found no difference in decomposition rates of single species grass litter with different N content. In this study, no differences in decomposition rates between *E. nuttallii* plants were observed, despite the manipulated differences in both C:N ratio and phenolic content. Possibly, the lack of changes in decomposition rates of *E. nuttallii* of different quality in our study may be explained by a simultaneous increase in phenolic content and decrease in C:N ratio under shaded conditions. Alternatively, the changes in microbial community composition observed in our experimental setup during decomposition may have counteracted any changes in decomposition rates due to a high functional redundancy in their effects on macrophyte decomposition. As interspecific differences related to C:N:P stoichiometry in decomposition rates can be observed [3], intraspecific differences in C:N and phenolic content in our study were either not strong enough to elicit a response in decomposition rates, or these parameters are less important on an intraspecific level to drive decomposition rates. Whether any of these hypothesized processes are major causes of our experimental findings remains an open question and would be an interesting avenue for future research.

### Macrophyte Tissue Quality Altered Microbial Community Composition

Both nutrient and light treatments affected bacterial and fungal community composition in epiphytic biofilms, and the effects of the light treatment during the growth experiment continued during the following decomposition experiment. As no effects of experimental treatments on ASV diversity were observed (Table 1), these findings indicate an ASV (i.e., species) replacement in the bacterial community. It should be noted that during the growth experiment, attached algae were present on the macrophytes, and their biomass responses to the light treatments was similar as their host plants (Table S5). As attached algae can release exudates that affect microbial community composition and abundance [19], the observed changes in biofilm composition may be the result of both macrophytes and their attached algae. An earlier study on epiphytic bacterial communities on the macrophyte *Cabomba caroliniana* did not reveal any differences in ASV richness across a range of shading treatments [28]. The experimental effects of the light treatment persisted during macrophyte decomposition, as epiphytic bacterial ASV diversity and community composition differed significantly between light treatments. To our knowledge, this is the first demonstration of a continuation of the selecting effects of light on epiphytic microbial community composition after exposure.

Bacterial communities in epiphytic biofilms in all experiments were dominated by Pseudomonadota (formerly known as Proteobacteria), which are a diverse phylum of gram-negative bacteria commonly observed in freshwater ecosystems [25, 52]. In particular, the families Comamonadaceae and Methylophilaceae dominated the bacterial community composition during the growth experiment (Fig. S4). The first family is diverse and commonly observed as a dominant bacterial group in oxic freshwater habitats [32]. Methylophilaceae, on the other hand, are a phylogenetically small group of methylotrophic bacteria which can be associated with the turnover of single-carbon compounds from phytoplankton blooms [38], and their presence in macrophyte biofilms has been described previously [11, 21]. During the initial phase in the decomposition experiment, characterized by fast litter decomposition, the bacterial community composition was dominated by Comamonadaceae as well. When decomposition rates decreased and the recalcitrant fraction was reached, this community was replaced by a more diverse bacterial assemblage (Fig. S7). In particular, the Saprospiraceae family made up a larger proportion at the end of the decomposition experiment. This bacterial family is known for its role in the breakdown of complex organic compounds [29], and its presence in decomposition of recalcitrant litter is thus consistent. It is possible that the shift in the bacterial community on the decomposing macrophytes toward the Saprospiraceae indicates a beneficial selective environment for their proliferation.

The fungal community in our experiments mainly consisted of Saksenaeaceae (Figs. S5 and S8), a family within the Mucoromycota, of which *Saksenaea oblongispora* was the dominant species during macrophyte decomposition. In a laboratory setting, this species is able to grow on a large range of carbon sources compared to other fungi within the Mucoromycota [35]. Its hypothesized associations with decomposition processes are reinforced here by its presence during macrophyte decomposition in Lake Müggelsee. During the growth experiment, shading led to a significantly altered fungal community as the proportion of unknown fungal sequences increased and the relative abundances of Aspergillaceae, Mycosphaerellaceae, and Psathyrellaceae decreased (Fig. S5). These changes in fungal community composition were correlated with light-driven changes in macrophyte stoichiometry (Fig. 5). As there is an overall lack of data on the effects of litter stoichiometry on aquatic fungi [12], these results contribute to our collective understanding of the link between macrophyte stoichiometry and its associated fungal community composition.

## Conclusion

We conclude that changes in light availability, a common phenomenon in freshwater environments during processes, such as eutrophication and brownification, significantly affect *E. nuttallii* tissue quality and their epiphytic bacterial and fungal biofilm composition during macrophyte decomposition. These alterations in macrophyte tissue quality and their associated biofilm may indicate either a high functional redundancy of diverse epiphytic microbial communities or were simply not sufficient to illicit changes in macrophyte decomposition rates. Nonetheless, our findings indicate that C:N:P stoichiometry of litter material, rather than phenolic content, appears to be a crucial factor in shaping the microbial communities during decomposition.

## Supplementary Information

Below is the link to the electronic supplementary material.

Supplementary file1 (DOCX 543 KB)

## Data Availability

The data and R scripts that support the findings of this paper are publicly available within the DANS Data Station Life Sciences at https://dans.knaw.nl/en/life-sciences/, with the following identifier: 10.17026/LS/IKCDHS.
