# Supplementary material for: Light-Driven Changes in Macrophyte Tissue Quality Affect the Composition of Associated Microbial Communities
Source: Microb Ecol. 2025 May 23;88(1):52. doi: 10.1007/s00248-025-02546-9 (PMC12101996; doi:10.1007/s00248-025-02546-9)
Supplement: Supplementary file 1 — Supplementary file1 (DOCX 543 KB) [file 248_2025_2546_MOESM1_ESM.docx]

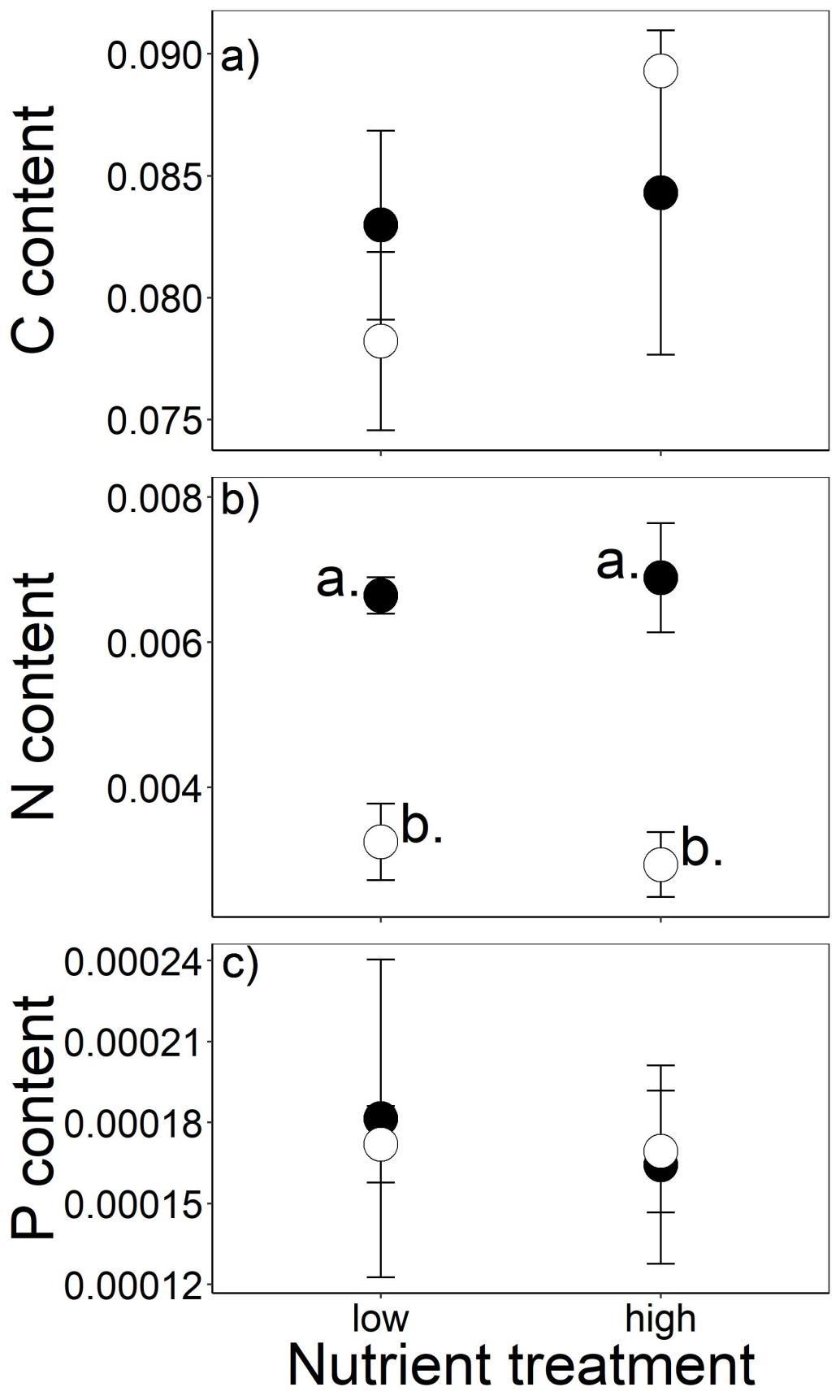


**Fig. S1**: Elemental composition of *Elodea nuttallii* grown in different sediment nutrient conditions, with a) carbon (C), b) nitrogen (N) and c) phosphorus (P) content, all depicted in mol/g dry weight (mean ± SE). Light treatments include full light (◌) and shaded (●) conditions and letters indicate significant pairwise differences.


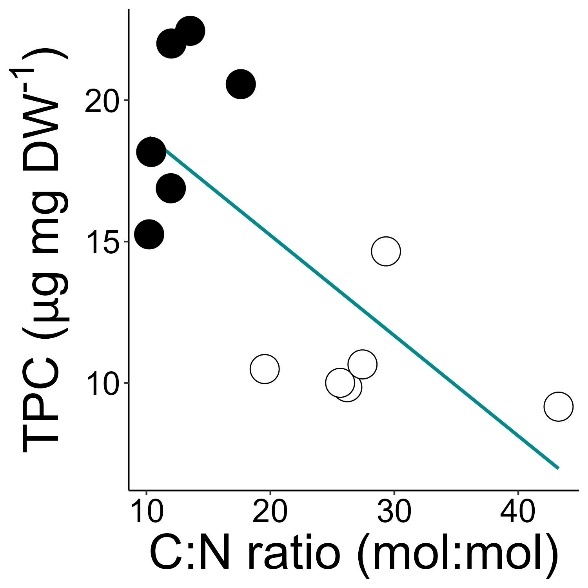


**Fig. S2:** Correlation between molar C:N ratio and total phenolic content (TPC) of aboveground biomass of *Elodea nuttallii*. Light treatments include full light (◌) and shaded (●) conditions.


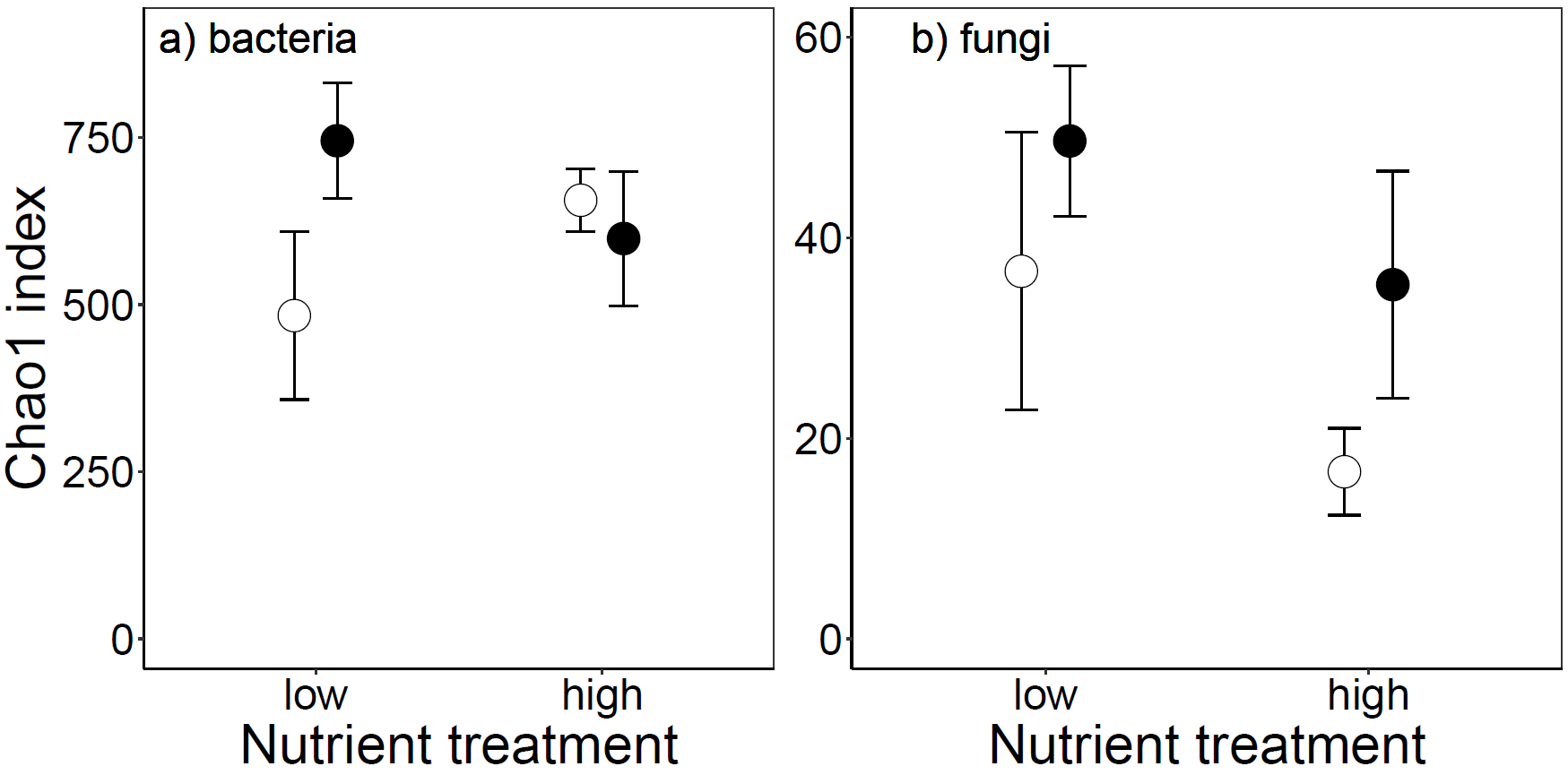


**Fig. S3:** Microbial alpha diversity, expressed as the bias-corrected Chao1 index (mean ± SE) on the freshwater macrophyte *Elodea nuttallii* grown at different sediment nutrient conditions, for a) bacterial and b) fungal communities. Light treatments include full light (◌) and shaded (●) condition.


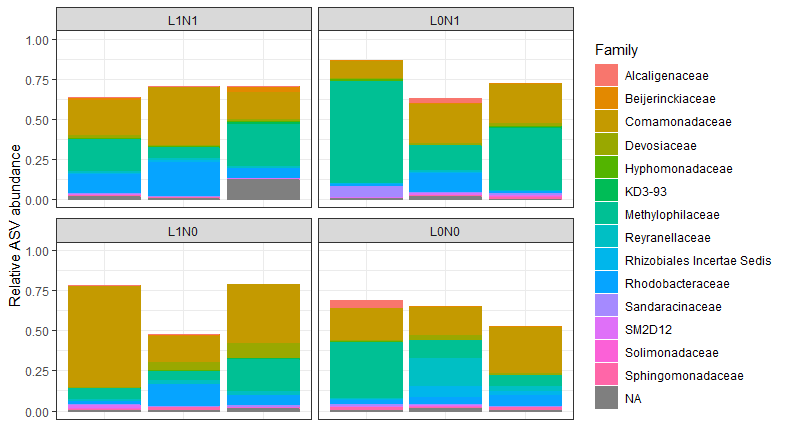


**Fig. S4**: Relative abundance of the bacterial families in the *Elodea nuttallii* growth experiment, based on the 50 most abundant 16S sequences. Each panel represents three replicates of an unique combination of nutrient and light treatment, with low (N0) and high (N1) nutrient treatments and shaded (L0) and full light (L1) treatments. Unknown sequences are indicated with NA.


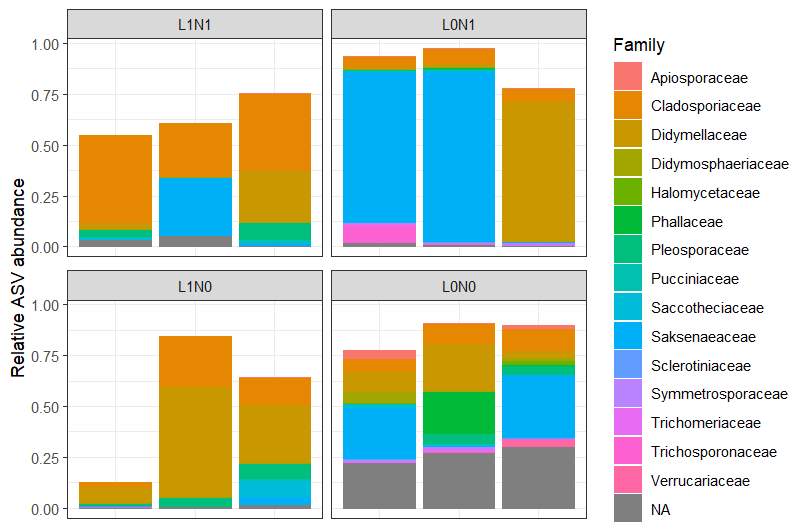


**Fig. S5**: Relative abundance of the fungal families in the *Elodea nuttallii* growth experiment, based on the 30 most abundant LSU sequences. Each panel represents three replicates of a unique combination of nutrient and light treatment, with low (N0) and high (N1) nutrient treatments and shaded (L0) and full light (L1) treatments. Unknown sequences are indicated with NA.

*
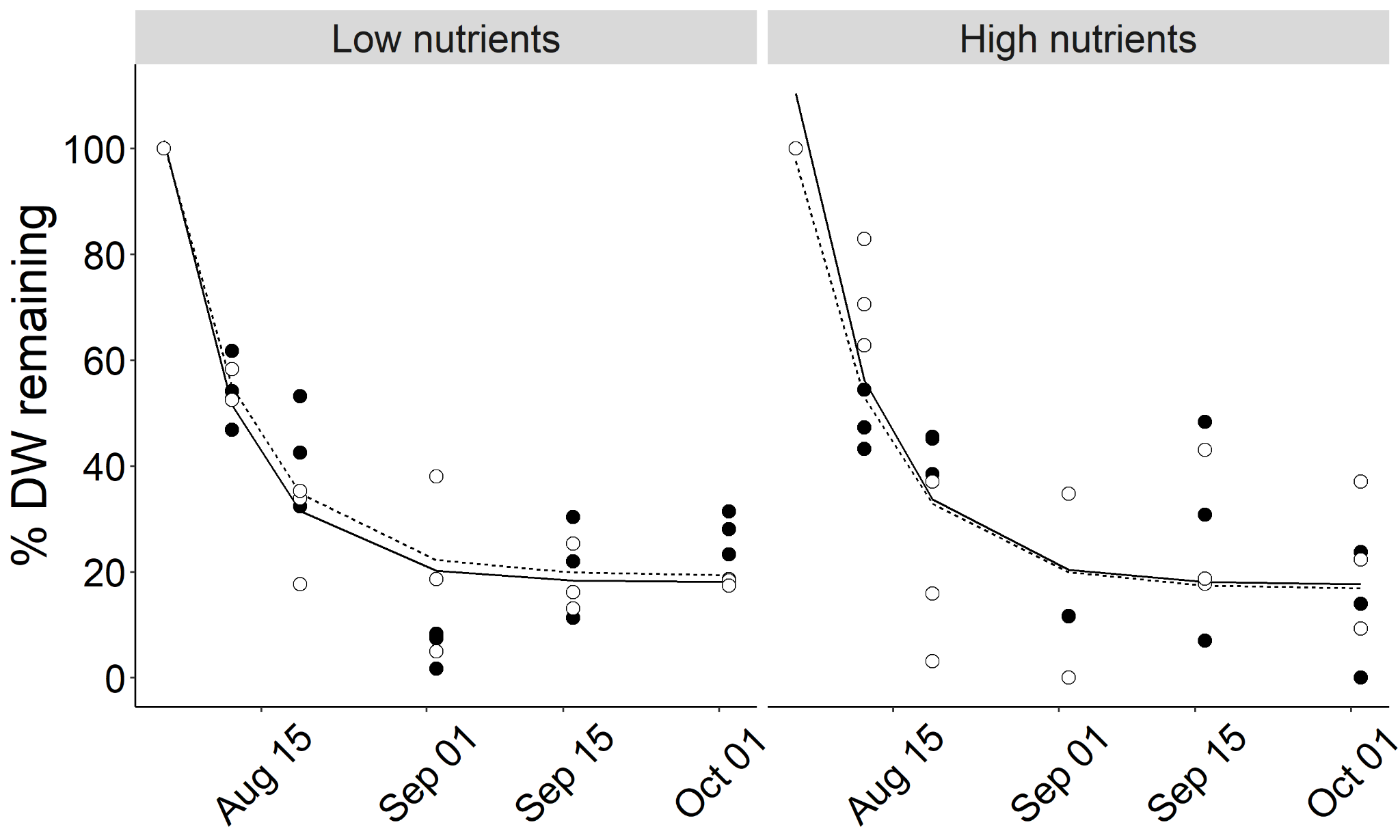
*

**Fig. S6**: Decomposition of *Elodea nuttallii* in eutrophic Lake Müggelsee in summer and autumn 2019, expressed as the percentage of biomass (dry weight: DW) remaining of macrophytes grown in full light (◌) and shaded (●) condition at a given sample time point. Fits of a two-phase decomposition model are indicated by the solid (full light) and dotted (shaded) lines.


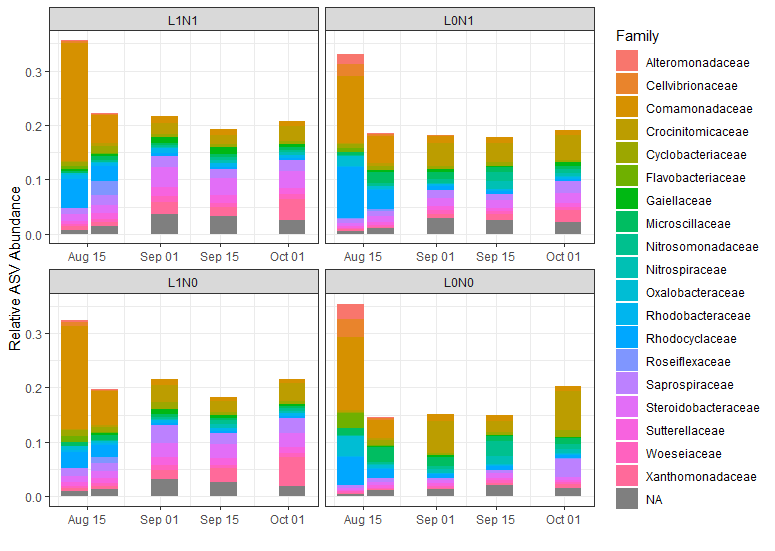


**Fig. S7**: Mean relative abundance of the bacterial families over time during decomposition of *Elodea nuttallii* in Lake Müggelsee, based on the 50 most abundant 16S sequences (N=3, except for L0N0 2019-09-02 and 2019-08-19, where N=2). Each panel represents a unique combination of nutrient and light treatment, with low (N0) and high (N1) nutrient treatments and shaded (L0) and full light (L1) treatments. Unknown sequences are indicated with NA.


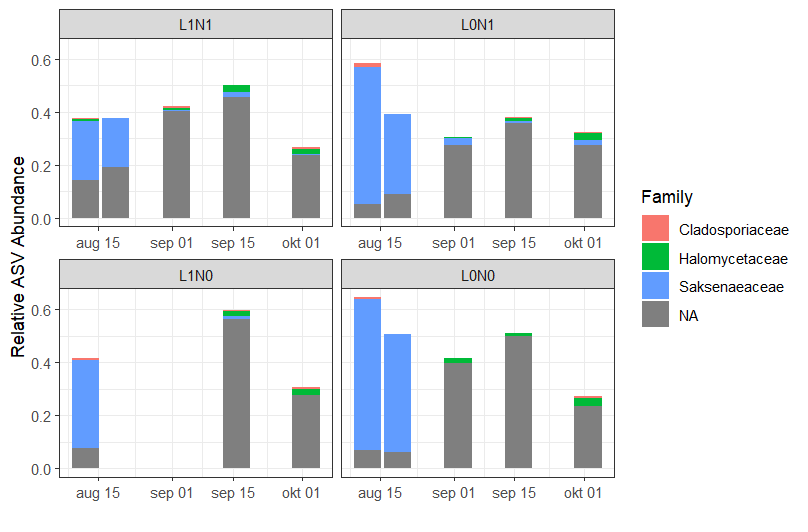


**Fig. S8**: Mean relative abundance of the fungal families over time during decomposition of *Elodea nuttallii* in Lake Müggelsee, based on the 50 most abundant LSU sequences (sample size varies between 1 and 3). Each panel represents a unique combination of nutrient and light treatment, with low (N0) and high (N1) nutrient treatments and shaded (L0) and full light (L1) treatments. Unknown sequences are indicated with NA.

**Table S1:** Detailed sediment information on experimental nutrient treatments, with Low (N0) and high (N1) nutrient treatments. Information is derived from the manufacturer (FloraSelf Vertriebs-GmbH, Oldenburg, Germany)

|  | **Unit** | **N0** | **N1** |
| --- | --- | --- | --- |
| Organic matter (OM) content | % | 2.5 | 25 |
| Nitrogen concentration | mg/L | 1 – 10 | 10 - 100 |
| Phosphorus concentration | mg/L | 0.2 – 2.2 | 2.2 – 21.8 |

**Table S2:** Primers used for Illumina Amplicon sequencing of the microbial community composition.

|  | **Sequence (5’to 3’)** | **Reference** |
| --- | --- | --- |
| Bacterial primers |  |  |
| 799F | AACMGGATTAGATACCCKG | (Chelius and Triplett 2001) |
| 1115R | AGGGTTGCGCTCGTTRC | (Reysenbach and Pace 1995) (cited in Baker et al. (2003) |
| Fungal primers |  |  |
| ITS4ngs (F) | CATATCAATAAGCGSAGGA | Modified reverse sequence of ITSngs from Tedersoo et al. (2015) |
| LF402 | TTCCCTTTYARCAATTTCAC | (Tedersoo et al. 2015) |

**Table S3**: Summary of % remaining reads (mean ± SD) after each step in the DADA2 bioinformatical pipeline.

|  | Bacterial 16S - experiment | Fungal LSU - experiment | Bacterial 16S - decomposition | Fungal LSU - decomposition |
| --- | --- | --- | --- | --- |
| Input | 100 | 100 | 100 | 100 |
| Filtered | 75.5 ± 4.3 | 55.3 ± 12.2 | 86.9 ± 3.9 | 87.0 ± 13.2 |
| Denoised Forward | 75.2 ± 4.3 | 54.7 ± 12.3 | 70.3 ± 11.1 | 82.7 ± 13.6 |
| Denoised Reverse | 75.3 ± 4.3 | 54.8 ± 12.3 | 86.8 ± 6.7 | 82.2 ± 13.9 |
| Merged paired-end reads | 73.9 ± 4.5 | 53.7 ± 13.3 | 56.9 ± 12.5 | 69.9 ± 13.7 |
| Chimeras removed | 72.8 ± 4.7 | 53.4 ± 12.2 | 49.4 ± 12.0 | 66.5 ± 13.0 |

**Table S4**: Accuracy of taxonomic identification during analysis. Given is the percent of sequence reads assigned to the different phylogenetic levels using the Silva v138.1 (bacteria) and Eukaryome v1.9.2 (fungi) databases.

|  | Bacterial 16S – growth experiment | Fungal LSU –  growth experiment | Bacterial 16S - decomposition | Fungal LSU - decomposition |
| --- | --- | --- | --- | --- |
| Kingdom | 99.6 | 88.7 | 100 | 94.3 |
| Phylum | 98.2 | 70.1 | 97.0 | 64.5 |
| Class | 96.4 | 70.0 | 93.3 | 62.0 |
| Order | 91.9 | 69.1 | 86.3 | 59.2 |
| Family | 83.0 | 62.3 | 72.1 | 42.3 |
| Genus | 57.6 | 59.0 | 40.8 | 38.1 |
| Species | 11.6 | 45.3 | 5.6 | 26.0 |

**Table S5**: Environmental conditions measured during both the growth and decomposition experiment of *Elodea nuttallii*. Values are mean (SD) and – indicates that that parameter was not measured.

|  | **Growth experiment** | | | | **Decomposition experiment** |
| --- | --- | --- | --- | --- | --- |
| **parameter** | **L0N0** | **L0N1** | **L1N0** | **L1N1** |  |
| Temperature (˚C) | 19.92 (2.33) | 19.67 (2.52) | 19.62 (2.26) | 19.41 (2.34) | 19.38 (2.65) |
| Oxygen (mg L^-1^) | 8.49 (0.94) | 7.51 (1.22) | 9.11 (1.42) | 8.28 (1.74) | 6.64 (1.32) |
| *p*H | 8.08 (0.10) | 7.75 (0.08) | 8.17 (0.24) | 7.97 (0.23) | 7.99 (0.24) |
| Phytoplankton (µg Chla L^-1^) | 0.10 (0.05) | 0.60 (0.44) | 11.79 (9.52) | 7.16 (6.85) | - |
| Attached algae (g DW) | 0.02 (0.03) | 0 (0) | 1.86 (1.33) | 0.57 (0.33) |  |
| Light availability (µmol m^-2^ s^-1^) | 29.6 (22.9) | 38.0 (25.9) | 148 (115) | 166 (122) | - |
| DOC (mg L^-1^) | - | - | - | - | 6.30 (0.45) |
| CO_2_ (µM) | - | - | - | - | 424 (352) |
| CH_4_ (µM) | - | - | - | - | 0.23 (0.21) |

**Supplementary methodology**

During the growth experiment, light availability was measured once a week between June 20^th^ and August 15^th^ with a spherical PAR sensor (QSPL2101, Biospherical Instruments, USA). *p*H was measured on June 20^th^ and 27^th^, using a WTW multimeter (Weilheim i.OB, Germany). Phytoplankton biomass, measured on August 17^th^, was approximated by chlorophyll fluorescence using a phyto-PAM. MiniDOT loggers (PME, USA) were deployed to measure oxygen and temperature continuously between June 27^th^ and August 15^th^.

During the decomposition experiment in Lake Müggelsee, *p*H, DOC, CO_2_ and CH_4_ were measured between August 5^th^ and October 2^nd^ (at the same time as litter bag sampling). *p*H was measured by a WTW multimeter (Weilheim i.OB, Germany) in-situ. DOC samples were filtered over prewashed GF/F filters and analyzed on a multi N/C 2100s instrument (Analytik Jena). Dissolved CO_2_ and CH_4_ were determined by equilibrating 500 ml water sample with 650 ml headspace through 1 minute vigorous shaking by a strong student. Gas concentrations in the headspace were analysed on a portable greenhouse gas analyser (GasScouter 4301, Picarro, USA). Temperature and oxygen were measured continuously throughout the same time period using miniDOT loggers.

**References**

Baker, G., J. J. Smith, and D. A. Cowan. 2003. Review and re-analysis of domain-specific 16S primers. Journal of microbiological methods **55**:541-555.

Chelius, M. K., and E. W. Triplett. 2001. The Diversity of Archaea and Bacteria in Association with the Roots of Zea mays L. Microbial Ecology **41**:252-263.

Reysenbach, A., and N. Pace. 1995. Reliable amplification of hyperthermophilic Archaeal 16S rRNA genes by the polymerase chain reaction.Thermophiles: A laboratory manual 101-105.

Tedersoo, L., S. Anslan, M. Bahram, S. Põlme, T. Riit, I. Liiv, U. Kõljalg, V. Kisand, H. Nilsson, F. Hildebrand, P. Bork, and K. Abarenkov. 2015. Shotgun metagenomes and multiple primer pair-barcode combinations of amplicons reveal biases in metabarcoding analyses of fungi. MycoKeys **10**:1-43.
